# Infant gut microbiota characteristics generally do not modify effects of lipid-based nutrient supplementation on growth or inflammation: secondary analysis of a randomized controlled trial in Malawi

**DOI:** 10.1038/s41598-020-71922-x

**Published:** 2020-09-09

**Authors:** Riley L. Hughes, Charles D. Arnold, Rebecca R. Young, Per Ashorn, Ken Maleta, Yue-Mei Fan, Ulla Ashorn, David Chaima, Chikondi Malamba-Banda, Mary E. Kable, Kathryn G. Dewey

**Affiliations:** 1 Department of Nutrition, University of California, Davis, CA USA; 2 Center for Child Health Research, Faculty of Medicine and Health Technology, Tampere University, Tampere, Finland; 3 Department of Pediatrics, Tampere University Hospital, Tampere, Finland; 4 College of Medicine, University of Malawi, Blantyre 3, Malawi; 5 School of Public Health and Family Medicine, University of Malawi College of Medicine, Blantyre, Malawi; 6 Immunity and Disease Prevention, Western Human Nutrition Research Center, Agricultural Research Service, USDA, Davis, CA USA

**Keywords:** Applied microbiology, Policy and public health in microbiology, Epidemiology, Paediatric research

## Abstract

An unhealthy gut microbial community may act as a barrier to improvement in growth and health outcomes in response to nutritional interventions. The objective of this analysis was to determine whether the infant microbiota modified the effects of a randomized controlled trial of lipid-based nutrient supplements (LNS) in Malawi on growth and inflammation at 12 and 18 months, respectively. We characterized baseline microbiota composition of fecal samples at 6 months of age (n = 506, prior to infant supplementation, which extended to 18 months) using 16S rRNA gene sequencing of the V4 region. Features of the gut microbiota previously identified as being involved in fatty acid or micronutrient metabolism or in outcomes relating to growth and inflammation, especially in children, were investigated. Prior to correction for multiple hypothesis testing, the effects of LNS on growth appeared to be modified by *Clostridium* (p-for-interaction = 0.02), *Ruminococcus* (p-for-interaction = 0.007), and Firmicutes (p-for-interaction = 0.04) and effects on inflammation appeared to be modified by *Faecalibacterium* (p-for-interaction = 0.03) and *Streptococcus* (p-for-interaction = 0.004). However, after correction for multiple hypothesis testing these findings were not statistically significant, suggesting that the gut microbiota did not alter the effect of LNS on infant growth and inflammation in this cohort.

## Introduction

Results of nutrient supplementation trials in undernourished populations have been mixed, particularly with regard to child linear growth outcomes^1–4^. As many of these studies address relevant nutrient deficiencies in these populations, this suggests that there may be a barrier to improvement in growth outcomes. One such potential barrier may be the gut microbiota of these individuals. Recent research suggests that the gut microbiome influences host response to diet (i.e. acts as an effect modifier)^5–11^Therefore, infants with an underdeveloped microbiota configuration may not respond in terms of improved growth or health outcomes to nutrient supplementation. If this is the case, then interventions should focus on improving the composition of the gut microbiota in these populations before, or in synergy with, nutrient supplementation, as suggested by Gehrig et al.^12^, as a sort of probiotic or prebiotic adjuvant^13,14^.

A growing body of research has documented effect modification of dietary interventions on metabolic outcomes by the gut microbiota^15,16^. However, very little is known about the potential of the microbiome to modify the effect of dietary interventions on growth and health outcomes in an undernourished or infant population. Several components of this question have been the subject of recent studies^2,12,17–19^ investigating the infant microbiota, its association with infant health outcomes, and modification of this community by dietary components. However, the question of whether characteristics of the baseline microbiota modify the effect of a nutritional intervention on infant health outcomes has not been addressed. Therefore, the objective of these analyses was to determine whether features of the infant gut microbiome at six months of age modify the effects of lipid-based nutrient supplements on child growth or inflammation in the iLiNS-DYAD-Malawi randomized controlled trial^20^.

Lipid-based nutrient supplements (LNSs) are formulated with essential fatty acids and numerous micronutrients, such as vitamin D and calcium^21^. The gut microbiome is predicted to affect host lipid metabolism^22^ and therefore likely impacts the effects of lipid supplementation. Of particular importance in the infant gut microbiota are bifidobacteria and lactobacilli. These taxa are among the earliest and most dominant^23–26^ colonizers of the infant gut and are involved in metabolism of α-linolenic acid (ALA)^27–34^ and micronutrients, including absorption of vitamin D and calcium^35–37^. These taxa are also involved in barrier function and protection against pathogenic bacteria^38–41^. Low abundance of these taxa has been associated with increased risk of developing allergies or atopy^42–47^ and studies have shown that supplementation with bifidobacteria or lactobacilli decreases the risk of allergy development and influences host plasma lipid composition in response to ALA supplementation^33,34^, though it must be recognized that effects may be specific to certain species of these bacterial taxa^48^.

In undernourished infant populations, *Faecalibacterium prausnitzii*, *Dorea*, *Clostridium,* and *Ruminococcus* have been associated with infant age, weaning-phase or health, while *Enterococcus*, *Escherichia*, and *Streptococcus* have been shown to be enriched in children with severe acute malnutrition (SAM) relative to healthy children^2,12,17^. Composite characteristics of human gut microbiota composition have also been associated with health outcomes in infants and adults. These include the microbiota-for-age z-score (MAZ), diversity and richness, the *Prevotella*/*Bacteroides* (P/B) ratio^49–52^, *Firmicutes*/*Bacteroidetes* (F/B) ratio, and the *Enterobacteriaceae*/*Bacteroidaceae* (E/B) ratio. MAZ was developed to assess microbiota development and maturity in undernourished populations of children^2^. This score is a composite of 24 bacterial taxa associated with chronologic age, thus providing a measure of maturity in the process of postnatal microbiota assembly.

While healthy infants have lower microbial diversity relative to adults^24^, lower microbiota diversity among infants has been associated with increased risk of food allergy and atopic eczema^53–55^. Ratios of taxa have also been found to be important in predicting health outcomes. For example, later development of food allergy has been associated with an elevated E/B ratio in infants^19^, suggesting that this pattern of microbiota composition in the infant may affect immune function and inflammation. The P/B and F/B ratios have been shown to be relevant to dietary response and health outcomes in adults^50–52,56,57^, though the effects of these patterns may not translate to different dietary interventions or to infants as the gut microbiota changes with age^58^. Therefore, it is of interest to investigate the effects of broad patterns of microbiota composition on response to a dietary intervention in addition to the effects of specific taxa.

In the iLiNS-DYAD-Malawi trial, LNS was provided to women during pregnancy and the first six months postpartum, and to their infants from 6 to 18 months of age. In the two control groups, women received iron and folic acid (IFA) during pregnancy only or multiple micronutrients (MMN) during pregnancy and the first six months postpartum, but their infants did not receive any supplement. The effects of the intervention on infant growth^20^, characteristics of the child’s gut microbiome^59^, and maternal breast milk composition^60^ have been published previously, as well as analyses that relate aspects of gut microbiome composition to infant growth and inflammation^61^, morbidity^62^, and environmental factors^63^. This analysis examines whether variability in infant gut microbiota composition, such as the ratios of taxa described above, may contribute to variability in response to the intervention.

## Results

### Study profile and characteristics of participants

**Figure 1:**
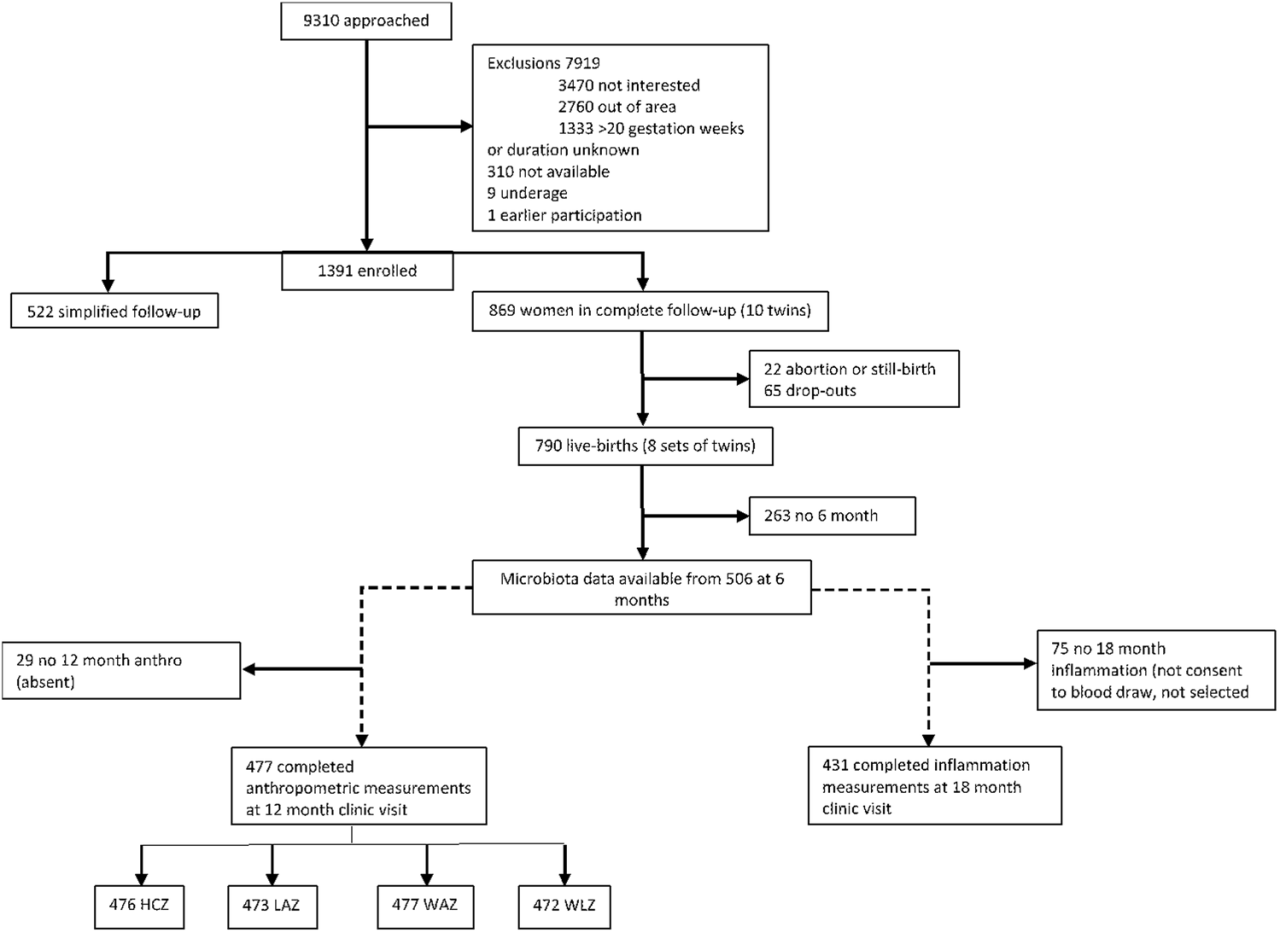
Participant flow. Participant flow in CONSORT-recommended format. AGP: alpha 1-acid glycoprotein, CONSORT: Consolidated Standards of Reporting Trials, CRP: C-reactive protein, HCZ: head circumference z-score, LAZ: length-for-age z-score, WAZ: weight-for-age z-score, WLZ: weight-for-length z-score.

For the current analysis, only participants who provided infant fecal samples at 6 months were included. Of the 790 live births from women selected to continue supplementation until 6 months postpartum, 506 infants had gut microbiota data available at the 6 month time point (Fig. 1). The 488 participants included herein were those with microbiota data at 6 months as well as at least one of the outcome variables for growth or inflammation. Characteristics of each of the intervention groups are shown in Table 1. The two control groups (IFA and MMN) groups were combined for these analyses. There were no significant group differences in maternal baseline characteristics, child sex, infant growth status at 6 months (length-for-age z-score (LAZ), weight-for-age z-score (WAZ), weight-for-length z-score (WLZ), head circumference z-score (HCZ)), inflammation (C-reactive protein (CRP) and alpha 1-acid glycoprotein (AGP)) at 6 months, dietary variables at 6 months, or baseline microbiota characteristics at 6 months. Table 2 also compares the baseline characteristics of participants included and excluded from these analyses. There were statistically significant differences in some characteristics: on average, the women included had lower body mass index (BMI), fewer education years completed, higher food insecurity, and lower household asset scores, and their infants had a less negative WLZ at 6 months, compared to those excluded.

**Table 1:** Characteristics of participants.

	LNS	Non-LNS	*p *Value	Total included	Excluded	*p* Value
Participants, n	167	348		515	354	
**Maternal**						
Age at enrollment, years†	25.0 ± 5.9	25.4 ± 6.5	0.515	25.2 ± 6.3	24.1 ± 6.1	0.008
Height, cm†	156.7 ± 5.8	156.1 ± 5.6	0.291	156.3 ± 5.7	155.8 ± 5.9	0.163
BMI, kg/m^2†^	21.8 ± 2.3	22.0 ± 2.8	0.496	21.9 ± 2.7	22.3 ± 3.0	0.101
Education completed, years†	3.2 ± 2.9	3.3 ± 3.2	0.939	3.2 ± 3.1	4.1 ± 3.6	< 0.001
Positive malaria RDT‡	21.1	22.3	0.764	21.9	24.6	0.353
HIV + ‡	15.0	11.2	0.227	12.4	9.7	0.238
**Household**						
Food insecure‡	40.0	40.1	0.980	40.1	34.6	0.118
Access to flushing toilet‡	9.6	11.3	0.576	10.7	9.3	0.523
Household asset score, z-score†	− 0.12 ± 0.96	− 0.06 ± 0.96	0.502	− 0.08 ± 0.96	0.21 ± 1.13	< 0.001
**Child**						
Female‡	47.3	53.2	0.214	51.3	56.2	0.192
LAZ at 6 mo†	− 1.54 ± 1.10	− 1.52 ± 1.14	0.819	− 1.53 ± 1.13	− 1.55 ± 1.16	0.780
WAZ at 6 mo†	− 0.76 ± 1.15	− 0.77 ± 1.09	0.898	− 0.77 ± 1.11	− 0.92 ± 1.17	0.109
WLZ at 6 mo†	0.00 ± 1.12	− 0.02 ± 1.03	0.795	− 0.01 ± 1.06	− 0.20 ± 1.12	0.047
HCZ at 6 mo†	− 0.63 ± 0.94	− 0.64 ± 1.00	0.958	− 0.63 ± 0.98	− 0.93 ± 1.08	0.001
High AGP at 6 mo‡	65.0	64.1	0.864	64.4	64.3	0.983
High CRP at 6 mo‡	28.3	27.9	0.939	28.0	34.0	0.173
Breastfeeding at 6 mo‡	100	99.1	0.999	99.4	98.2	0.182
Predominantly breastfeeding*‡	13.1	17.7	0.234	16.2	17.3	0.772
Consumed maize porridge*‡	87.9	93.2	0.062	91.5	89.0	0.398
Consumed animal- source food*‡	66.7	73.3	0.148	71.1	71.3	0.965
*Bifidobacterium* (above median)‡	52.3	49.0	0.496			
*Lactobacillus* (above median)‡	54.2	48.1	0.206			
*Clostridium* (above median)‡	51.0	49.9	0.818			
*Dorea* (above zero)‡	65.2	62.5	0.565			
*Enterococcus* (above median)‡	49.0	51.4	0.633			
*Escherichia* (above median)‡	63.9	63.1	0.863			
*Faecalibacterium* (above zero)‡	53.0	61.9	0.061			
*Ruminococcus* (above median)‡	47.8	53.2	0.266			
*Streptococcus* (above median)‡	54.8	47.7	0.145			
*Firmicutes* (above median)‡	49.7	50.2	0.923			
*Bacteroidetes* (above median)‡	52.3	49.0	0.496			
*Enterobacteriaceae* (above median)‡	47.74	51.35	0.458			
*Bacteroidaceae* (above median)‡	47.74	51.05	0.496			
Shannon Diversity†	1.59 ± 0.60	1.59 ± 0.56	0.981			
Chao1†	218.7 ± 140.9	229.7 ± 144.8	0.080			
MAZ†	0.66 ± 2.34	0.48 ± 2.30	0.345			

*Dietary practices were assessed between 5 and 7 months of age. Predominant breastfeeding is defined as consuming only breastmilk and/or water-based liquids. Porridge and animal source food prevalence defined by consumption in the previous 24 h.

^†^Values for intervention groups shown as mean ± standard deviation.

^‡^Values for intervention groups shown as percent.

**Table 2:** Change in z-scores from 6 to 12 months of age and inflammation status at 18 months of age, by intervention group.

	LNS	Non-LNS	p-value	Difference in means (95% CI)
ΔLAZ 6–12 mo†	− 0.20 ± 0.76	− 0.30 ± 0.70	0.18	0.10 (− 0.05,0.25)
ΔWAZ 6–12 mo†	− 0.22 ± 0.67	− 0.24 ± 0.60	0.77	0.02 (− 0.11,0.15)
ΔWLZ 6–12 mo†	− 0.48 ± 0.86	− 0.39 ± 0.83	0.28	0.09 (− 0.26,0.08)
ΔHCZ 6–12 mo†	− 0.39 ± 0.58	− 0.42 ± 0.57	0.62	0.03 (− 0.09,0.14)
CRP 18 mo*‡	0.85 (− 0.50,2.08)	0.71 (− 0.45, 1.92)	0.81	1.05 (− 0.37,0.47)
AGP 18 mo*‡	0.21 (− 0.13, 0.56)	0.26 (− 0.03, 0.48)	0.89	1.01 (− 0.09,0.08)

*Analyzed with log transformed values, consequently the difference in means is back-transformed and represents the ratio of the means.

^†^Values for intervention groups shown as mean ± standard deviation.

^‡^Values for intervention groups shown as median and interquartile range.

There were no significant differences between intervention groups in the changes in z-scores between 6 and 12 months or mean CRP and AGP values at 18 months (Table 2).

### Effect modification

The rationale for the specific aspects of the gut microbiome examined for potential effect modification is presented in Supplementary Table 1. Because maternal supplementation did not alter the gut microbiota composition at 6 months, the 6 month infant gut microbiota composition can be considered the “baseline” for the purpose of the analyses herein. We hypothesized that among infants with higher abundances of *Bifidobacterium* and *Lactobacillus* and higher diversity and richness as well as lower E/B and F/B ratios and MAZ scores at 6 months, LNS would have a positive effect on growth and would be associated with reduced inflammation, whereas among infants with lower abundances of *Bifidobacterium* and *Lactobacillus* and low diversity and richness as well as higher E/B and F/B ratios and MAZ scores, these effects would not be observed. Additional microbiota taxa chosen as potential effect modifiers were those found in two or more relevant publications^2,12,17^ to be age- or health-discriminatory: *Clostridium*, *Dorea*, *Enterococcus*, *Escherichia*, *Faecalibacterium*, *Ruminococcus*, and *Streptococcus*. As all of these analyses are exploratory, statistical significance of the interactions between potential effect modifiers and the LNS intervention is discussed both before and after correction for multiple hypothesis testing.

**Figure 2:**
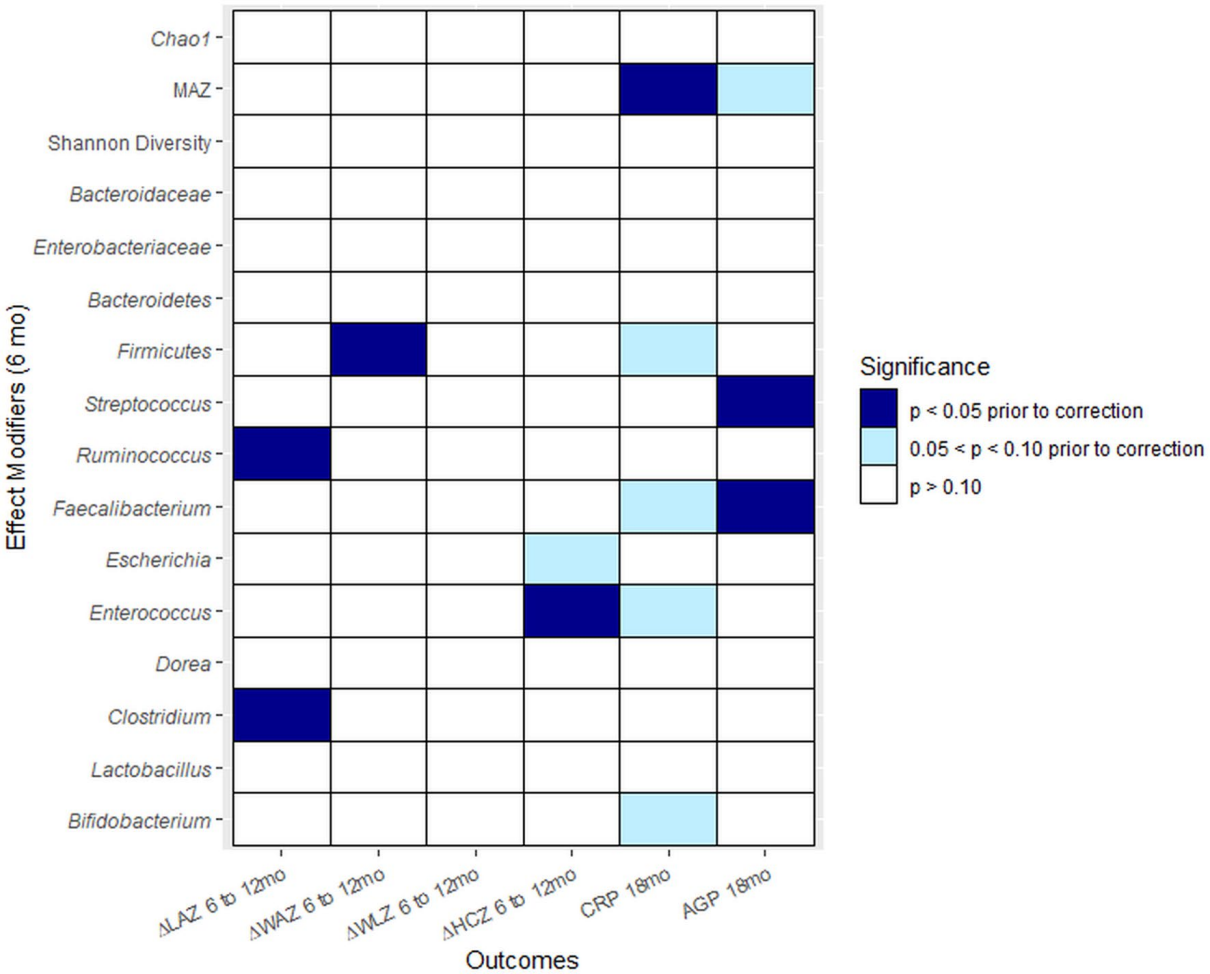
Heatmap of p-values for interaction. Heatmap displays levels of p-values for interactions with the LNS intervention. No p-values were less than 0.10 after correction for multiple hypothesis testing.

**Figure 3:**
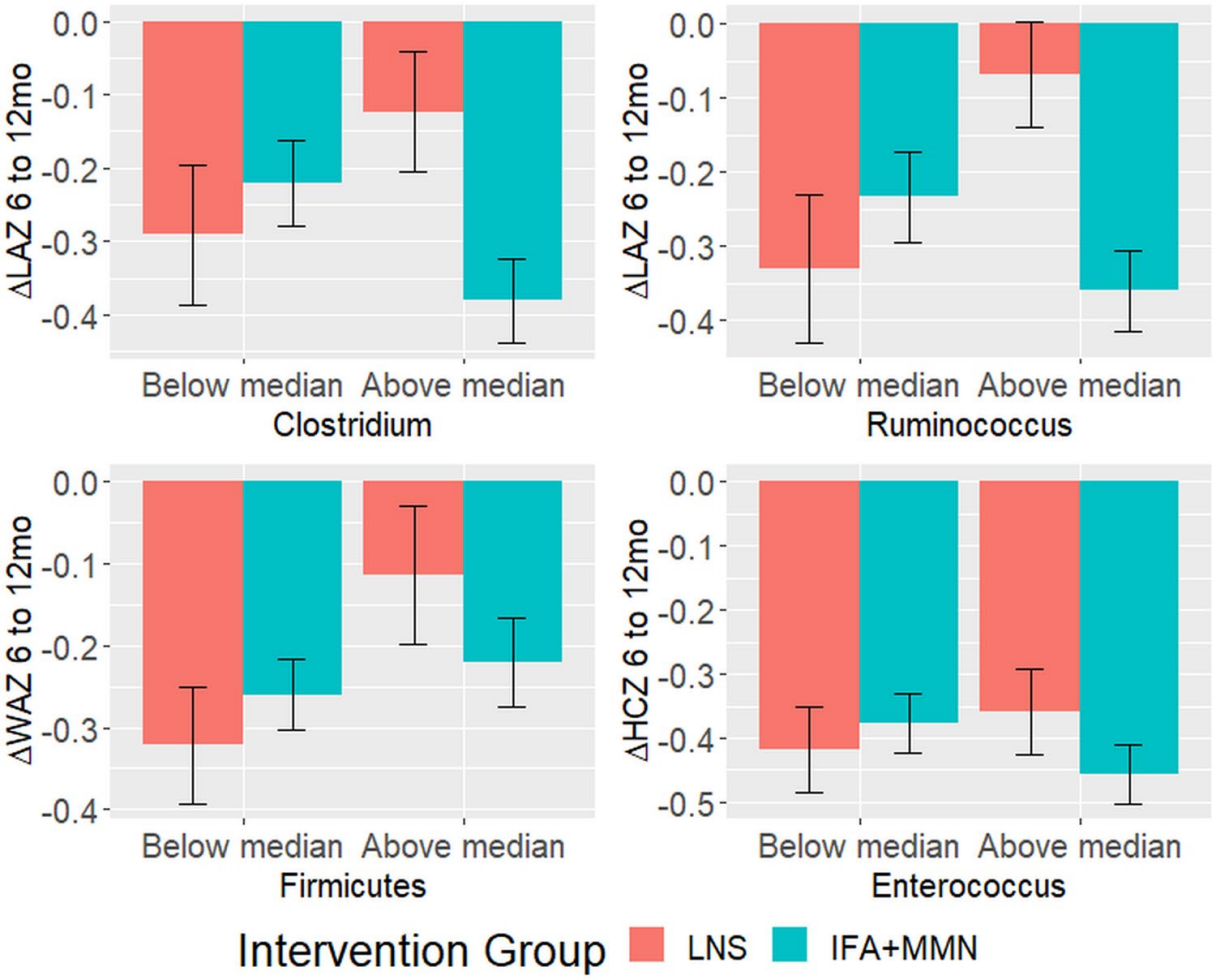
Growth outcomes exhibiting effect modification by 6 month gut microbiota characteristics, regarding the effect of LNS on change in z-scores from 6 to 12 months of age. Data shown are for effect modification interactions for which the p-value was < 0.05 before correction for multiple hypothesis testing.

There was no evidence of effect modification on growth outcomes for the following characteristics of the gut microbiome: *Bifidobacterium*, *Lactobacillus*, E/B ratio (or *Enterobacteriaceae* or *Bacteroidaceae* separately), *Bacteroidetes*, *Dorea*, *Faecalibacterium*, *Escherichia*, *Streptococcus*, Shannon index, Chao1, or MAZ (Fig. 2). Abundances of individual taxa were calculated as percent relative abundances. Effect modification relationships for the infant growth outcomes that were significant before correction for multiple hypothesis testing are shown in Fig. 3. Among infants with *Clostridium* relative abundance above the median (0.47%) and *Ruminococcus* abundance above the median (0.02%) at 6 months, there was a less negative change in LAZ from 6 to 12 months in the LNS group relative to the non-LNS group, whereas among infants with *Clostridium* and *Ruminococcus* relative abundance below the median at 6 months, there was little difference between the LNS and non-LNS groups (p-for-interaction_Clo_ = 0.02, p-for-interaction_Rum_ = 0.007). Among infants with *Firmicutes* abundance above the median (18.5%) at 6 months, there was a less negative change in WAZ from 6 to 12 months in LNS relative to non-LNS infants, whereas the opposite was seen among those with abundance below the median (p = 0.04). Among infants with *Enterococcus* abundance above the median (0.16%) at 6 months, there was a slightly less negative change in HCZ from 6 to 12 months in LNS relative to non-LNS infants, whereas the opposite was seen among those with abundance below the median (p = 0.03).

**Figure 4:**
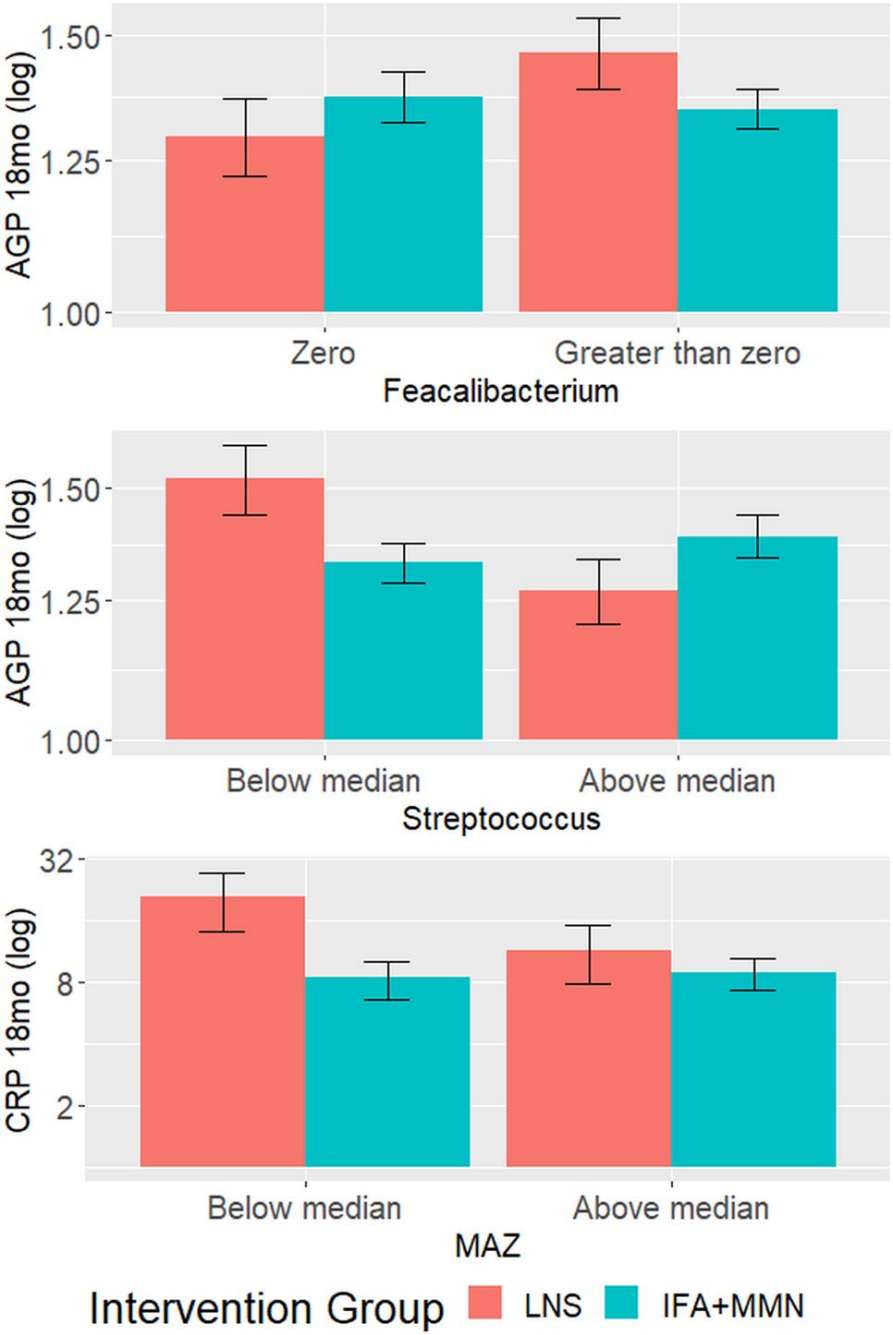
Effect modification by 6 month gut microbiota characteristics, regarding the effect of LNS on inflammation at 18 months of age. Data shown are for effect modification interactions for which the p-value was < 0.05 before correction for multiple hypothesis testing.

There was no evidence of effect modification on inflammation outcomes for the following characteristics of the gut microbiome: *Bifidobacterium*, *Lactobacillus*, E/B ratio (or *Enterobacteriaceae* or *Bacteroidaceae* separately), *Firmicutes*, *Bacteroidetes*, *Clostridium*, *Ruminococcus*, *Enterococcus*, *Dorea*, *Escherichia*, Shannon index, or Chao1 (Fig. 2). Effect modification relationships for the inflammation outcomes that were significant before correction for multiple hypothesis testing are shown in Fig. 4. Among infants with *Faecalibacterium*, AGP was higher in LNS than non-LNS infants, whereas the opposite was true for infants without *Faecalibacterium* (p-for-interaction = 0.03). Conversely, among infants with *Streptococcus* above the median (3.87%), AGP at 18 months was lower in LNS relative to non-LNS infants, while the opposite was true for infants with *Streptococcus* abundance below the median (p-for-interaction = 0.004). Lastly, among infants with MAZ above the median (0.24), CRP was lower in LNS relative to non-LNS infants, whereas among infants with MAZ below the median, CRP was higher in LNS than non-LNS infants (p-for-interaction = 0.007).

In the adjusted models for the interactions between the above effect modifiers and intervention group, no interactions remained statistically significant after false-discovery rate (FDR) correction using the Benjamini–Hochberg Procedure.

## Discussion

Previous studies have shown a link between gut microbial taxa and healthy infant growth, and the authors posit that identifying complementary foods that target the developing infant gut microbiome is crucial to developing effective treatments for malnutrition^2,12,17^. To our knowledge, the current analysis is the first attempt to assess whether characteristics of the infant gut microbiota actually modify the effects of a nutritional intervention on growth or inflammation.

Prior to correction for multiple hypothesis testing, our results suggest that *Clostridium* and *Ruminococcus* may modify the effect of the SQ-LNS intervention on change in LAZ from 6 to 12 months. Both *Clostridium* and *Ruminococcus* are fiber-fermenting bacteria^36^, so higher levels of these taxa in the gut could indicate higher fiber intake, such as from maize porridge^64^. Greater intake of fiber and complementary foods could contribute either to the infants’ potential to benefit from LNS supplementation (i.e., higher maize porridge consumption, and potentially lower breast milk intake, may be a risk factor for morbidity or high phytate intake) or their potential to respond to LNS supplementation (i.e., infants were eating more in general, so could respond to the micronutrients in LNS). Unfortunately, in this sample we were unable to adequately assess this hypothesized dietary relationship because only limited food frequency questionnaire data were available. *Firmicutes* abundance above the median also appeared to modify the effect of the LNS intervention on change in WAZ from 6 to 12 months before correction for multiple hypothesis testing. *Firmicutes* and *Actinobacteria* are initial colonizers of the infant gut that are well suited to utilize both breast milk and non-digestible plant polysaccharides^65^. *Firmicutes* has been shown to be positively correlated with weight, in children and adults, which may be linked to increased production of short-chain fatty acids (SCFAs) that can be used for energy^66–68^. As SCFAs were not measured in this population, it is unknown whether this might explain why the effect of LNS on change in WAZ was more positive among infants with higher relative abundance of *Firmicutes*.

Before correction for multiple hypothesis testing, *Faecalibacterium* and *Streptococcus* appeared to modify the effects of the LNS intervention with respect to AGP at 18 months, though in opposite directions: among infants with *Faecalibacterium*, the LNS group had higher AGP compared to non-LNS infants, while among infants with *Streptococcus* above the median, the LNS group had lower AGP compared to non-LNS infants. *Faecalibacterium* is both an acetate consumer and butyrate producer that may contribute to gut barrier integrity^69^ and has been associated with positive growth outcomes in similar child populations^2,12,70^. Thus, it is possible that when *Faecalibacterium* was not present, infants did not benefit from its role in gut barrier integrity, so LNS may have compensated for this and reduced AGP. In infants who had *Faecalibacterium*, there was no advantage of LNS. Certain species of *Streptococcus* may be beneficial^71^, while others have been associated with conditions such as SAM^12^. As our analysis did not have sufficient sequencing depth to determine species-level classification, we were not able to determine which species or strains were present. Lastly, MAZ appeared to modify the effect of LNS on CRP. However, because CRP is a short-term marker of inflammation, it is unclear how MAZ at 6 months may modify the effect of LNS on this outcome at 18 months.

The above relationships were not significant after correction for multiple hypothesis testing. The lack of significant effect modification in the current dataset could be related to the fact that there was no significant main effect of the intervention on growth and inflammation outcomes in this cohort, though there was in other cohorts^72,73^. Therefore, these questions should be investigated in populations in which positive effects of LNS on growth and inflammation have been demonstrated.

One limitation of the current analysis is that it did not include metagenomic or metatranscriptomic data. It is possible that it is the functional capacity of the microbial community, or species-specific taxa rather than genus-level taxonomic composition, that would be more likely to enhance or limit the effects of a nutritional intervention. Future studies examining effect modification would benefit from both high-resolution taxonomic and functional analyses of the gut microbiota. Such information is needed for different populations, for different markers of response, and for different nutritional interventions. Additionally, differences between included and excluded participants indicate that the results of this analysis may not be generalizable to the rest of the population.

Although we found no conclusive evidence of effect modification in this analysis, the relationships observed before correction for multiple hypothesis testing may be worth additional investigation. Specifically, further research on the potential of taxa such as *Clostridium*, *Ruminococcus*, *Faecalibacterium*, *Streptococcus*, and *Firmicutes* to modify the effects of nutrient supplementation on infant growth and inflammation would be useful. Such research will contribute to our evolving understanding of the nexus between diet, the gut microbiota, and health.

## Methods

### Study design and outcomes

A randomized, controlled, partially blinded, parallel-group clinical trial known as the International Lipid-based Nutrient Supplements DYAD (iLiNS-DYAD) trial was conducted in the Mangochi district of rural Malawi^1,74^. The main study hypothesis was that children whose mothers were provided with LNS during pregnancy and for 6 months after delivery and who themselves received LNS from 6 to 18 months of age would have a higher mean length at 18 months than children whose mothers received either IFA during pregnancy only or MMN supplementation during pregnancy and lactation and who themselves received no LNS. For primary outcome analysis and study information, please refer to Ashorn et al.^74^.

The enrollment to the study took place in one public district hospital (Mangochi), one semiprivate hospital (Malindi), and 2 public health centers (Lungwena and Namwera) in Mangochi District, southern Malawi. The target population comprised pregnant women who came for antenatal care at any of the study clinics during the enrollment period and met the inclusion criteria^75^. Between February 2011 and August 2012, the iLiNS team members approached a total of 9,310 women, from whom 1,391 (14.9%) were enrolled in the trial and were randomly assigned to 1 of the 3 intervention groups. Of these, 869 women were assigned to the complete intervention and follow-up until 18 months after delivery. Singleton children born to these women formed the sample for the present study. Infants born to the remaining 522 women who were assigned to pregnancy intervention only were not included in the present analyses (Fig. 1). Details of the study, including the original sample size calculation, are described elsewhere^75^.

At enrollment, study personnel collected data on socio-demographic status, maternal age, height, body mass index (BMI), parity, education, HIV status, hemoglobin concentration, household assets, food security, source of drinking water, access to sanitary facilities, and season. Household asset and food security indices were created as previously described^76,77^. Details of the trial are available at the National Institutes of Health (USA) clinical trial registry (www.clinicaltrials.gov), under the registration number NCT01239693 (11/10/2010). The trial was conducted in adherence with the Good Clinical Practice guidelines and ethical standards of the Helsinki Declaration. The trial protocol was approved and ethical clearance to conduct the study was granted by the University of Malawi College of Medicine Research and Ethics Committee (COMREC) and the ethics committee at Tampere University Hospital District, Finland. Informed consent was obtained from each participant before being enrolled into the study. An independent data safety and monitoring board monitored the incidence of suspected serious adverse events during the trial.

Women were randomly assigned to three groups as described previously^74^: iron and folic acid during pregnancy only (IFA), a multiple micronutrient (MMN) tablet during pregnancy and the first 6 months postpartum, or LNS during pregnancy and the first 6 months postpartum. Briefly, an independent researcher (not involved with the trial) created individual randomization slips in blocks of 9. The slips were then packed in sealed, numbered, and opaque randomization envelopes stored in numerical order. Enrolled women were asked to choose 1 of the top 6 envelopes in the stack, and the contents of each chosen envelope indicated her participant number and group allocation. A statistician not involved in the study maintained the intervention code, which was not broken until all laboratory and statistical analyses of primary outcomes were performed. The IFA/placebo and MMN capsules were identical in appearance.

Children born to mothers in the IFA and MMN groups received no supplementation; children in the LNS group received small quantity lipid-based nutrient supplementation (SQ-LNS) from 6 to 18 months. The LNS given to infants differed from that given to mothers as the nutrient content was designed to meet the needs of infants^21^.

### Stool sample collection and sequencing

All stool sample collection and sequencing occurred prior to the current data analysis. Sample collection and sequencing was performed as previously described^61^. Briefly, infant stool samples were collected at 6 months, 12 months, and 18 months of age. Stool samples were collected in the home, the morning of the clinic visits and frozen at − 20 °C before being transported to the central clinic in Mangochi and stored at − 80 °C. After DNA extraction, the variable region 4 (V4) of bacterial 16S rRNA was amplified by PCR and sequenced using Illumina MiSeq. QIIME 1 was used to cluster reads into operational taxonomic units (OTUs) at 97% sequence identity using the May 2013 Greengenes database^78^. Taxonomy was assigned using the Ribosomal Database Project classifier 2.4.

Raw counts were rarefied to 10,000 reads as determined by construction of a rarefaction curve and singleton OTUs were filtered out. Abundance counts were normalized using total sum scaling (TSS).

### Outcomes and effect modifiers

All outcomes were assessed as continuous variables. This included LAZ, WAZ, WLZ, HCZ, CRP, and AGP. Age- and sex-standardized anthropometric indices (LAZ, WAZ, WLZ, and HCZ) were calculated using the WHO Child Growth Standards^79^. Growth outcomes were assessed as the change from 6 to 12 months of age while inflammatory outcomes were assessed as absolute values at 18 months of age.

Potential effect modifiers chosen for the analysis included relative abundance of *Bifidobacterium, Lactobacillus*, *Clostridium*, *Dorea*, *Enterococcus*, *Escherichia*, *Faecalibacterium*, *Ruminococcus*, and *Streptococcus* as well as patterns of microbiota composition including E/B ratio, F/B ratio, α-diversity (Shannon index), richness (Chao1), and MAZ (Supplementary Table 1). The E/B and F/B ratios were analyzed separately by their component parts (e.g. *Enterobacteriaceae* and *Bacteroidaceae; Firmicutes* and *Bacteroidetes*) since we observed abundances below the detectable limit of taxa which made the ratios incalculable for 33% and 12% of children, respectively.

Dichotomous variables were created to categorize taxonomic features into values above and below the median for all taxa except *Bifidobacterium*, which displayed a normal distribution. This allowed us to uniformly handle the skew of microbiota measures. For taxa for which the median was zero, such as *Dorea* and *Faecalibacterium*, we categorized into presence or absence, or abundance below the detectable limit.

### Statistical analysis

Statistical analyses were performed in R (version 3.4.0)^80^.

Maternal IFA and MMN intervention groups were combined into one control group because neither supplement was provided to the child and neither contained fats. Inflammation outcomes, AGP and CRP, were natural log transformed.

Potential effect modifiers were assessed with an interaction term between the effect modifier variable and intervention group in covariate adjusted ANCOVA or logistic models. Potential adjustment covariates included site of enrollment, estimated pre-pregnancy maternal BMI, maternal height, maternal education, maternal age, maternal HIV status, delivery method, parity, household assets, food security index, and season at 6 months postpartum and were included if significantly associated with the outcome at 10% level of significance in a bivariate analysis. We controlled for multiple hypothesis testing using Benjamini–Hochberg corrections, using a 0.15 false discovery rate as the threshold for significance.

## Supplementary information

Supplementary information
