# Supplementary material for: Infant gut microbiota characteristics generally do not modify effects of lipid-based nutrient supplementation on growth or inflammation: secondary analysis of a randomized controlled trial in Malawi
Source: Sci Rep. 2020 Sep 9;10:14861. doi: 10.1038/s41598-020-71922-x (PMC7481312; doi:10.1038/s41598-020-71922-x)
Supplement: Supplementary file 1 — Supplementary information [file 41598_2020_71922_MOESM1_ESM.docx]

**Infant gut microbiota characteristics generally do not modify effects of lipid-based nutrient supplementation on growth or inflammation: secondary analysis of a randomized controlled trial in Malawi**

**Riley L. Hughes^1^, Charles D. Arnold^1^, Rebecca R. Young^1^, Per Ashorn^2,3^, Ken Maleta^4^, Yue-Mei Fan^2^, Ulla Ashorn^2^, David Chaima^5^, Chikondi Malamba-Banda^5^, Mary E. Kable^6^, Kathryn G. Dewey^1*^**

^1^Department of Nutrition, University of California, Davis, CA, USA

^2^Center for Child Health Research, Tampere University, Faculty of Medicine and Health Technology, Tampere, Finland

^3^Department of Pediatrics, Tampere University Hospital, Tampere, Finland

^4^College of Medicine, University of Malawi, Blantyre 3, Malawi

^5^School of Public Health and Family Medicine, University of Malawi College of Medicine, Blantyre, Malawi

^6^Immunity and Disease Prevention, Western Human Nutrition Research Center, Agricultural Research Service, USDA, Davis, CA

*kgdewey@ucdavis.edu

# Supplementary Table 1. Effect modifier metrics

| Variable | Associations in the literature |
| --- | --- |
| *Bifidobacterium* | Improved barrier function [17, 19, 20]  ALA metabolism [24-29]  Micronutrient metabolism [31-33]  Future weight gain in children [11]  Reduced risk of allergies/atopy [22, 23] |
| *Lactobacillus* | Improved barrier function [18, 19]  ALA metabolism [25, 26]  Micronutrient metabolism [32, 33]  Reduced risk of allergies [21] |
| *Enterobacteriaceae*/*Bacteroidaceae* (E/B) ratio | Increased risk of food allergies [12] |
| *Firmicutes*/*Bacteroidetes* (F/B) ratio | Increased risk of food allergies [41] |
| Alpha Diversity (Shannon index) | Reduced risk of food allergies and atopic eczema [42-44] |
| Richness (Chao1) | Reduced risk of food allergies [12] |
| Microbiota-for-age z-score (MAZ) | Calculated as described previously [5] and also as previously calculated by the Gordon lab (<https://gordonlab.wustl.edu/>). |
| *Clostridium, Dorea*, *Faecalibacterium*, *Ruminococcus* | Associated with infant age, weaning-phase, or health [2, 13, 19] |
| *Enterococcus*, *Escherichia*, *Streptococcus* | Derived from children with severe acute malnutrition (SAM) [2, 13, 19] |

476 HCZ

473 LAZ

477 WAZ

472 WLZ
